# Elevated atmospheric CO_2_ concentrations alter grapevine (*Vitis vinifera*) systemic transcriptional response to European grapevine moth (*Lobesia botrana*) herbivory

**DOI:** 10.1038/s41598-019-39979-5

**Published:** 2019-02-28

**Authors:** Annette Reineke, Moustafa Selim

**Affiliations:** Geisenheim University, Department of Crop Protection, Von-Lade-Str. 1, D-65366 Geisenheim, Germany

## Abstract

Atmospheric carbon dioxide (CO_2_) concentrations are among the chief factors shaping the mode and magnitude of interactions between plants and herbivorous insects. Here, we describe the first global analysis of systemic transcriptomic responses of grapevine *Vitis vinifera* plants to feeding of European grapevine moth *Lobesia botrana* larvae at future elevated CO_2_ concentrations. The study was conducted on mature, fruit-bearing grapevine plants under ambient and elevated CO_2_ concentrations in a grapevine free-air carbon dioxide enrichment (FACE) facility. Grapevine transcriptional response to herbivory was clearly dependent on phenological stage, with a higher number of differentially expressed genes identified at fruit development compared to berry ripening. At fruit development, more transcripts were differentially expressed as a response to herbivory under elevated compared to ambient CO_2_ concentrations. Classification of the respective transcripts revealed that in particular genes involved in metabolic pathways, biosynthesis of secondary metabolites and plant-pathogen interactions were significantly enriched. Most of these genes had similar expression patterns under both CO_2_ concentrations, with a higher fold-change under elevated CO_2_ concentrations. Differences in expression levels of a subset of herbivory responsive genes were further validated by RT-qPCR. Our study indicates that future elevated CO_2_ concentrations will affect interactions between grapevine plants and one of its key insect pests, with consequences for future relevance of *L. botrana* in worldwide viticulture.

## Introduction

Plants interact with herbivorous insects in complex and multi-faceted ways^1–4^. Abiotic conditions prevailing in the respective environment are among the chief factors influencing the mode and magnitude of these interactions. In particular, temperature^5,6^, plant water status^7,8^ and atmospheric carbon dioxide (CO_2_) concentrations^9,10^ have been shown to shape the defence responses of plants and thus the extent of foliage consumed by herbivorous insects. Accordingly, rising global surface temperatures coupled with elevated CO_2_ concentrations as well as alterations in amount and extremity of precipitation or drought events as predicted under future climate change scenarios^11,12^ will greatly contribute to the scale and direction of these interactions. However, not all plant-insect-systems respond identically to shifts in the respective abiotic parameters. For example, an increase in atmospheric CO_2_ concentration has been shown to decrease chemical resistance in the legume *Medicago truncatula* resulting in an increased growth rate of the pea aphid (*Acyrthosiphon pisum*)^13^. At the same time, plants grown under elevated atmospheric CO_2_ concentrations often have lower tissue nitrogen concentrations resulting from a dilution due to the accumulation of non-structural carbohydrates^14^. Insect herbivores, in turn, need to compensate for this dilution effect by increasing consumption of foliage to cover their nitrogen demands^15,16^. This has e.g. been shown for maize (*Zea mays*) and Asian corn borer (*Ostrinia furnacalis*), where a CO_2_-mediated lower nitrogen content and higher C:N ratio and thus a decrease in plant nutritional quality caused a significant decline in insect survival and weight gain as well as an altered larval food consumption^17^. Growth of gypsy moth (*Lymantria dispar*) larvae was significantly inhibited by elevated CO_2_ and CO_2_-induced changes in quality of leaves of both poplar (*Populus pseudo-simonii*) and birch (*Betula platyphylla*)^18^. Similarly, population density and body mass of vine weevils (*Otiorhynchus sulcatus*) feeding on roots of black currant (*Ribes nigrum*) decreased under elevated CO_2_^19^. Moreover, production of plant hormones like ethylene or jasmonic acid is suppressed by increasing CO_2_, while salicylic acid levels have been shown to increase at the same time, affecting specific secondary chemical pathways involved in transcriptional regulation of specific plant defence-related genes^15^. Accordingly, a general statement on the effects of global climate change on plant-insect interactions, future extents of herbivorous leaf damages and putative reductions in crop yields cannot be made and have to be assessed for each plant-insect system and each particular feeding guild^20^.

Grapevine (*Vitis* spp.) is an important global commodity crop, which is planted throughout temperate regions worldwide. As a perennial cropping system often cultivated for several decades, grapevine is particularly prone to changes in climatic conditions, which can modulate the plant’s transcriptional and metabolic profile, stress responses and accordingly affect plant vegetative and reproductive development. For example, prolonged drought as expected for several viticultural regions under future climate change has been shown to alter grape berry fruit secondary metabolism with potential effects on grape and wine antioxidant potential, composition, and sensory features^21^. Moreover, heat stress affects metabolic pathways linked to berry composition^22,23^ as well as net carbon budget^24^. At the same time, vineyards are habitats to a variety of arthropod pests, which are affected by the same abiotic conditions as the plant itself. A recent review by Reineke and Thiéry^25^ summarizes the effects of climate change on both grapevine as a host plant for phytophagous insects, as well as on grape insect pests and their natural enemies. Yet, so far nothing is known on grapevine’s response to insect herbivory under future climatic conditions.

The European grapevine moth (*Lobesia botrana*, Den. & Schiff., Lepidoptera: Tortricidae) is regarded as one of the major insect pests of grapevine in Europe. It is a multivoltine species occurring in at least two generations, with larvae of the first generation feeding on grapevine flowers (anthophagous generation) and those of the second and following generations feeding on berries (carpophagous generation) at different ripening stages^26,27^. Accordingly, larvae of the anthophagous generation reduce number of flowers and fruit set, those of the carpophagous generation cause significant yield loss and increase the incidence of Botrytis and other secondary fungi causing grape bunch rot. Recently, European grapevine moth has also been shown to have a high invasive potential, as it was accidentally introduced into South America and California, where it spread rapidly across vineyards^28^. Moreover, European grapevine moth abundance and accordingly pest pressure is expected to rise under future climate change due to an earlier appearance of adults in spring, an increased number of generations and thus a prolonged season to interact with its host plant^25,29^.

In the present study, we carried out the first global analysis of transcriptomic response of grapevine plants to feeding of a herbivorous insect at two different phenological grapevine stages. Moreover, it is the first assessment of systemic responses in leaves of field-grown, mature and fruit-bearing plants under ambient (current) and elevated (future) CO_2_ concentrations, grown in a grapevine free-air carbon dioxide enrichment (FACE) facility, via high throughput sequencing of transcriptomes (RNA-Seq). We were particularly interested in answering the following questions: (1) Is the same set of genes expressed after *L. botrana* herbivory at two different grapevine phenological stages? (2) Do grapevine plants show differential transcriptomic responses to *L. botrana* herbivory under ambient and elevated CO_2_ concentrations? (3) How do grapevine plants respond to elevated CO_2_ concentrations under *L. botrana* herbivory? This study will thus provide first insights into the genome-wide transcriptional responses of grapevine plants to feeding of a herbivorous insect, both under current and future CO_2_ concentrations. It will therefore also indicate the future importance of the European grapevine moth as a pest insect for worldwide viticulture.

## Results

### Transcriptome sequencing (RNA-Seq) dataset

RNA sequencing of 24 grapevine leaf samples generated an average of 15,420,000 raw paired-end reads (reads with a length of 101 bp) for each sample, covering about 1.6 Gbp of sequencing raw data (Supplementary Table S1). Raw paired-end data have been deposited in the National Center for Biotechnology Information (NCBI) under BioProject ID PRJNA417047 and Sequence Read Archive under accession numbers SAMN08093445-SAMN08093492 (Supplementary Table S1). After trimming and quality filtering to remove adapters and low-quality data, between 4.9 to 18.4 million clean paired end reads for each sample were obtained (Supplementary Table S1) with an average of 48% GC content.

In order to map cDNA fragments obtained from RNA sequencing, *V. vinifera* GCF_000003745.3 was used as a reference genome. The overall read mapping ratio (total number mapped reads / total number processed reads) ranged from 52 to 86% (Supplementary Table S1).

From a total of 28,936 genes, 14,173 genes had a FPKM value of 0 in more than one of the 24 samples and were therefore excluded, resulting in 14,763 genes which were used for further analysis.

### Grapevine transcriptional response to *L. botrana* herbivory

**Figure 1:**
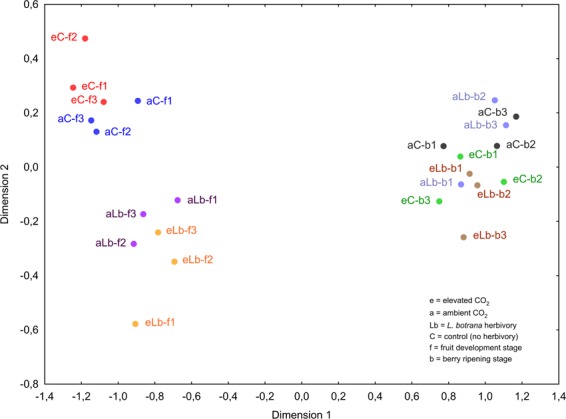
Multidimensional scaling (MDS) analysis of grapevine RNA-Seq profiles coloured according to CO_2_ concentration, *L. botrana* herbivory and grapevine growth stage. Euclidean distance was used to measure between samples dissimilarities over gene expression values. Each dot represents an RNA pool of three biological replicates obtained from one VineyardFACE ring. Blue = non-infested control plants at ambient CO_2_ and growth stage fruit development; red = non-infested control plants at elevated CO_2_ and growth stage fruit development; purple = *L. botrana*-infested plants at ambient CO_2_ and growth stage fruit development; orange = *L. botrana*-infested plants at elevated CO_2_ and growth stage fruit development; black = non-infested control plants at ambient CO_2_ and growth stage berry ripening; green = non-infested control plants at elevated CO_2_ and growth stage berry ripening; light blue = *L. botrana*-infested plants at ambient CO_2_ and growth stage berry ripening; brown = *L. botrana*-infested plants at elevated CO_2_ and growth stage berry ripening.

Gene expression levels for grapevine plants at two different growth stages (fruit development and berry ripening), at two different levels of CO_2_ concentrations (ambient CO_2_ (aCO_2_) and elevated CO_2_ (eCO_2_)) as well as for non-infested control plants and plants exposed to *L. botrana* herbivory were analysed by multivariate analysis to determine how well the gene expression profiles distinguished between the sampling time points and CO_2_ factors. In a multidimensional scaling plot (MDS; Fig. 1) three clusters are formed, which fit the time and herbivory sampling factors (stress = 0.029). The first dimension clearly separates samples taken at the first grapevine growth stage (fruit development) from those sampled at the later growth stage (berry ripening), thus explaining the largest proportion of variation in the given dataset. Grapevine leaf samples obtained at the fruit development stage are clearly separated along the second dimension by the factor *L. botrana* herbivory. In addition, MDS visualisation indicates that grapevine transcriptomes are subject to distinct changes according to the CO_2_ concentration at which the respective plants were grown.

### Differential expression of grapevine genes in response to *L. botrana* herbivory and elevated CO_2_ concentrations

**Figure 2:**
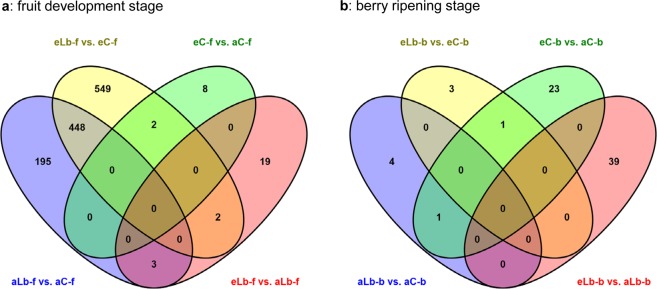
Venn diagram showing the number of significantly differentially expressed genes in grapevine plants at growth stages fruit development (**a**) and berry ripening (**b**). For each growth stage, number of genes differentially expressed in four pairs are shown, i.e. plants grown at elevated vs. ambient CO_2_ concentration without herbivory (eC vs. aC); plants grown at ambient CO_2_ with vs. without *L. botrana* herbivory (aLb vs. aC); plants grown at elevated CO_2_ concentration with vs. without *L. botrana* herbivory (eLb vs. eC); plants grown at elevated CO_2_ concentration vs. ambient CO_2_ with *L. botrana* herbivory (eLb vs. aLb).

Of a total of 14,763 genes used for differential expressed gene (DEG) analysis, no significant differences were found in gene expression levels as a response to *L. botrana* herbivory under ambient or elevated CO_2_ concentrations at two different growth stages when using *p*-values adjusted according to the Benjamini and Hochberg method. However, when less stringent parameters were considered by using unadjusted *p*-values, a substantial number was significantly differentially expressed in pairwise comparisons as a result of *L. botrana* herbivory over the two time points of sampling (fruit development and berry ripening) (Supplementary Table S3). Feeding of *L. botrana* larvae on grapevine plants grown at ambient CO_2_ concentrations resulted in 646 DEGs at the fruit development stage (aLb-f vs. aC-f), while in grapevine plants grown under elevated CO_2_ (eLb-f vs. eC-f) 1001 genes were differentially expressed as a result of herbivory (Table 1, Fig. 2). In addition, 448 DEGs were shared between plants grown at both CO_2_ concentrations (Fig. 2), representing those genes which were differentially expressed in grapevine plants as a response to *L. botrana* herbivory, irrespective of the CO_2_ concentration. At the fruit development stage, 24 DEGs were identified under *L. botrana* herbivory at eCO_2_ compared to aCO_2_ (eLb-f vs. aLb-f) of which 2 and 3 DEGs were shared with DEGs identified after herbivory at eCO_2_ and aCO_2_, respectively (Fig. 2). In non-infested control plants, 10 and 25 genes were differentially regulated in grapevine as a response to eCO_2_ at growth stages fruit development (eC-f vs. aC-f) and berry ripening (eC-b vs. aC-b), respectively (Table 1).

**Table 1:** Number of differentially expressed genes (DEGs, up- or downregulated) in grapevine plants as a response to *L. botrana* herbivory under ambient or elevated CO_2_ concentrations at two different growth stages.

Growth stage	Grapevine response to treatment	No. of DEGs		
		up	down	total
Fruit development	Response to *L. botrana* herbivory under aCO_2_ (aLb-f vs. aC-f)	388	258	646
	Response to *L. botrana* herbivory under eCO_2_ (eLb-f vs. eC-f)	491	510	1001
	Response to eCO_2_ (eC-f vs. aC-f)	3	7	10
	Response to eCO_2_ under *L. botrana* herbivory (eLb-f vs. aLb-f)	9	15	24
Berry ripening	Response to *L. botrana* herbivory under aCO_2_ (aLb-b vs. aC-b)	4	1	5
	Response to *L. botrana* herbivory under eCO_2_ (eLb-b vs. eC-b)	2	2	4
	Response to eCO_2_ (eC-b vs. aC-b)	10	15	25
	Response to eCO_2_ under *L. botrana* herbivory (eLb-b vs. aLb-b)	18	21	39

Genes were considered to be differentially expressed if they displayed a fold change ≥2 and an independent t-test raw *p*-value of < 0.05.

When *L. botrana* larvae fed on grapevine berries, which were ripe for harvest, only a small number of DEGs in leaves next to the feeding site were identified. Under ambient CO_2_ (aLb-b vs. aC-b) and elevated CO_2_ (eLb-b vs. eC-b) concentrations, *L. botrana* herbivory resulted only in 5 and 4 DEGs, respectively, with none of the genes shared between both groups (Table 1, Fig. 2). With *L. botrana* herbivory, 39 DEGs were identified at eCO_2_ compared to aCO_2_ (eLb-b vs. aLb-b) (Table 1, Fig. 2). At both growth stages (fruit development and berry ripening), none of the DEGs were shared in grapevine plants exposed to eCO_2_ and *L. botrana* herbivory (data not shown).

Taken together, at the fruit development stage, more DEGs were regulated under eCO_2_ compared to aCO_2_, indicating that grapevine plants show a CO_2_ effect in response to *L. botrana* herbivory at the level of gene expression, with a considerably stronger transcriptomic response under elevated eCO_2_ conditions.

### Response at growth stage fruit development

**Figure 3:**
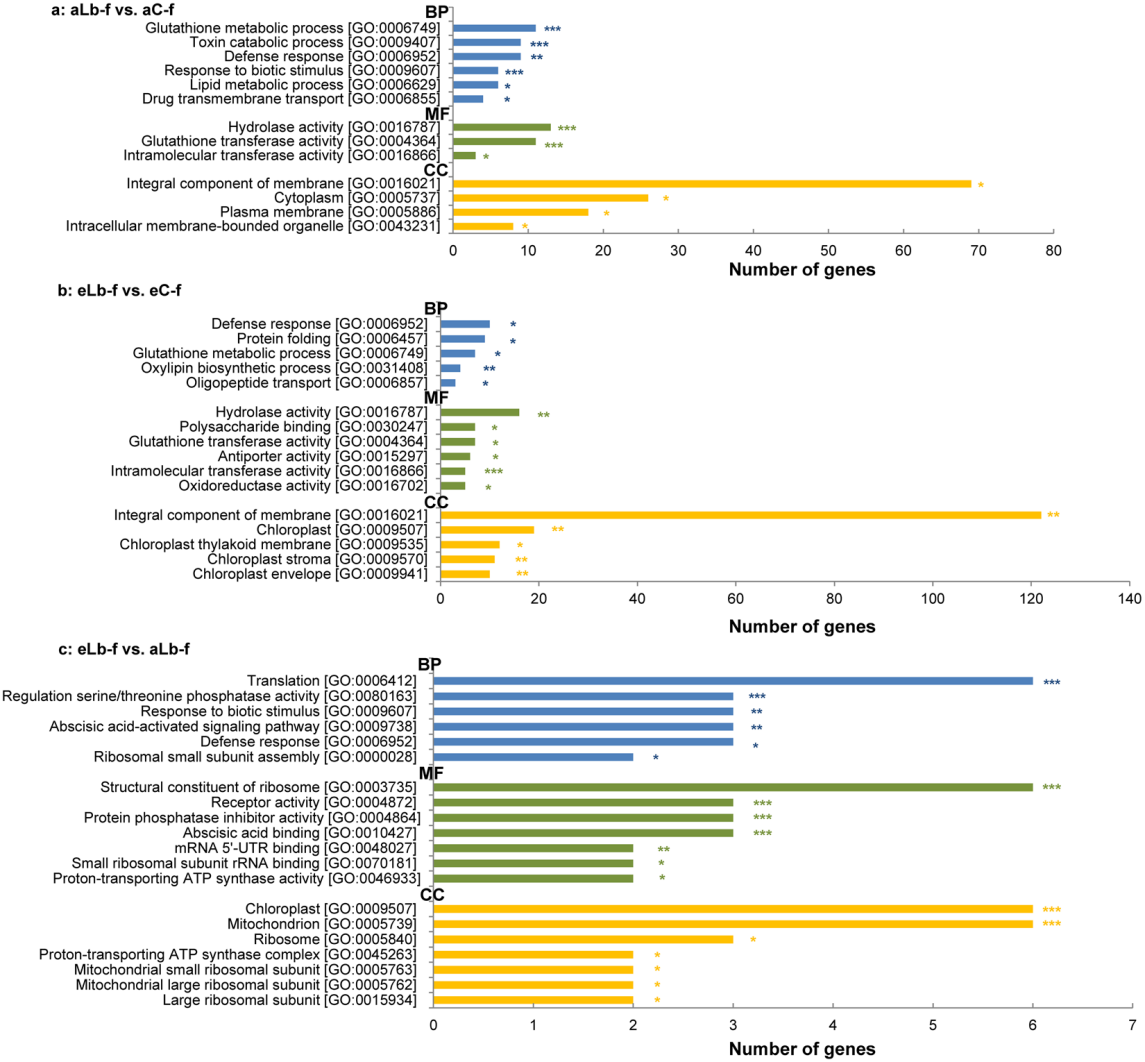
Enriched GO-terms (y axis labels) associated to DEGs as a response to *L. botrana* herbivory in grapevine plants at growth stage fruit development. (**a**) Response to herbivory under ambient CO_2_ (aLb-f vs. aC-f). (**b**) Response to herbivory under elevated CO_2_ (eLb-f vs. eC-f); (**c**) Response to elevated CO_2_ under herbivory (eLb-f vs. aLb-f). GO-term ontologies are coloured as blue = Biological Process (BP); green = Molecular Function (MF); yellow = Cellular Component (CC). Asterisks indicate significance at *p < 0.05, **p < 0.01 and ***p < 0.001.

GO enrichment analysis was used to identify the major gene groups affected by insect herbivory under both CO_2_ concentrations at grapevine fruit development. GO term analysis found six biological processes, three molecular functions as well as four cellular components that were significantly over-represented in response to *L. botrana* herbivory under aCO_2_ (Fig. 3a). Under eCO_2_, five biological processes, six molecular functions and five cellular components were significantly over-represented in response to *L. botrana* herbivory (Fig. 3b). As a response to elevated CO_2_ under herbivory six biological processes, seven molecular functions and seven cellular components were significantly over-represented (Fig. 3c). In particular, genes operating in processes involving glutathione metabolism, responses to biotic stimuli or defence responses were significantly enriched in grapevine plants in response to *L. botrana* herbivory at both aCO_2_ and eCO_2_ concentrations (Fig. 3a,b).

**Figure 4:**
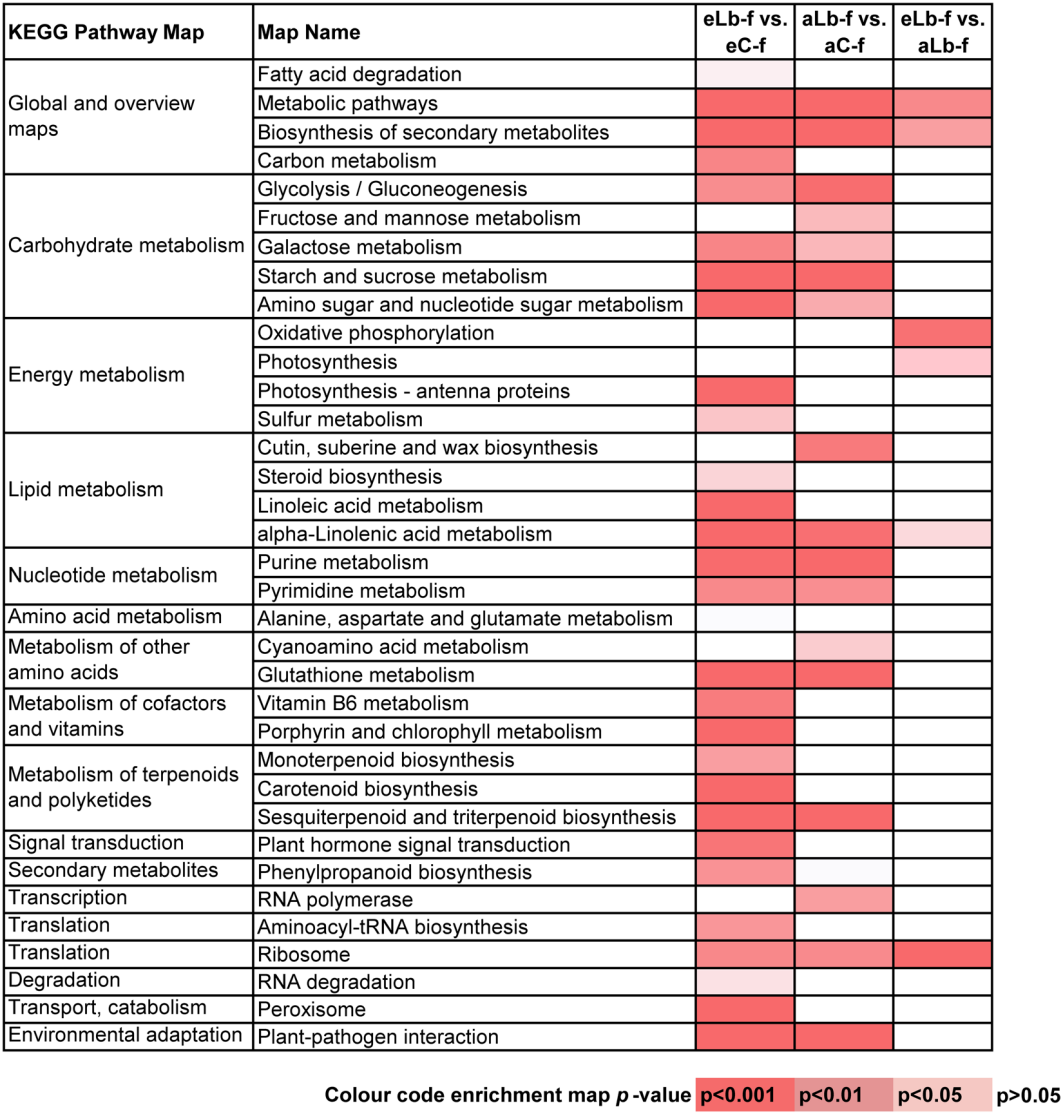
KEGG pathway classification of the grapevine transcriptome at the growth stage fruit development. Significantly enriched pathways as a response to *L. botrana* herbivory under eCO_2_ (eLb-f vs. eC-f) and aCO_2_ (aLb-f vs. aC-f) are shown as well as the effect of eCO_2_ on grapevine response to herbivory (eLb-f vs. aLb-f). Heatmap colour code represents significantly enriched pathways at different *p*-values.

To further investigate the biochemical pathways these DEGs are involved in they were mapped to terms in the KEGG database. For 1226 significantly differentially expressed genes identified at the grapevine growth stage fruit development (Fig. 2a), 197 different genes were assigned to a total of 35 KEGG pathways, which were significantly enriched (*p*-value < 0.05) (Fig. 4). The DEGs identified for eLb-f vs. eC-f and aLb-f vs. aC-f could be categorized into 29 and 18 significantly enriched pathways, respectively, while the DEGs identified for the comparison eLb-f vs. aLb-f were assigned to only six pathways (Fig. 4). Genes involved in primary metabolic pathways, biosynthesis of secondary metabolites and plant-pathogen interactions were the most significantly enriched.

Genes identified either via GO enrichment analysis or KEGG database mapping to have a function in processes like “plant-pathogen interaction”, “defence response” or “response to biotic stimuli” are of particular interest for understanding future grapevine responses to herbivory under elevated CO_2_. Details of these genes for grapevine growth stage fruit development are shown in Supplementary Table S2. Most of these genes have similar expression patterns as a result to *L. botrana* herbivory under ambient and elevated CO_2_ concentrations, respectively. For some genes, e.g. for the lipase-like PAD4 gene (GenBank Protein ID XP_002280786.1) or a calcium-dependent protein kinase (GenBank Protein ID XP_002267099.1), the overall fold change is however higher under elevated CO_2_. In contrast, e.g. the pathogenesis-related protein 10.8 (GenBank Protein ID XP_002273815.2) and a putative disease resistance protein (At1g50180; GenBank Protein ID XP_010658280.1) are significantly downregulated in grapevine plants as a response to *L. botrana* herbivory at elevated CO_2_ concentration (eLb-f vs. eC-f), while expression of both genes is not significantly affected by herbivory under ambient CO_2_ concentration (aLb-f vs. aC-f) (Supplementary Table S2).

### Response at growth stage berry ripening

In samples obtained at the growth stage of berry ripening, no GO terms were significantly over-represented as a response to *L. botrana* herbivory under ambient CO_2_ concentrations (aLb-b vs. aC-b). Under elevated CO_2_ (eLb-b vs. eC-b) one GO term identified as transcription factor activity (GO:0003700; represented by 2 genes; *p* = 0.0396) was significantly over-represented (Supplementary Table S3) as a response to herbivory. The same GO term was also significantly over-represented as a response to elevated CO_2_ under herbivory (eLb-b vs. aLb-b) (transcription factor GO:0003700; represented by 4 genes, *p* = 0.0028) as well as DNA binding activity (GO:0003677; represented by 4 genes, *p* = 0.017). The three genes functioning as ethylene-responsive transcription factors were categorized into both GO terms (GO:0003700 and GO:0003677; Supplementary Table S3). The few DEGs identified in grapevine leaves after herbivory at the growth stage of berry ripening were assigned to three KEGG pathways, which were however not significantly enriched (not shown).

### Validation of RNA-Seq data by quantitative reverse transcription-PCR (RT-qPCR)

**Figure 5:**
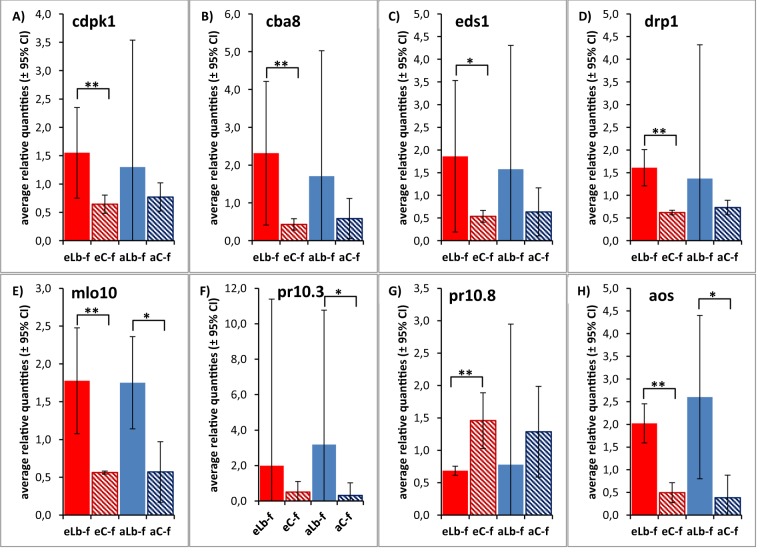
Expression of eight *L. botrana* herbivory responsive genes in grapevine plants at growth stage fruit development. Average relative fold expression (shown with the 95% confidence interval; *n* = 3) as a response to herbivory under elevated CO_2_ (eLb-f vs. eC-f) and ambient CO_2_ (aLb-f vs. aC-f) was assessed by RT-qPCR. (**A**) brassinosteroid insensitive 1-associated receptor kinase 1-like (cdpk1); (**B**) calcium-binding allergen Ole e 8-like (cba8); (**C**) enhanced disease susceptibility 1 (eds1); (**D**) disease resistance protein RPM1-like (drp1); (**E**) mildew resistance locus o 10 (mlo10); (**F**) pathogenesis-related protein 10.3 (pr10.3); (**G**) pathogenesis-related protein 10.8 (pr10.8); (**H**) allene oxide synthase (aos). Asterisks indicate significant differences in expression ratios at *p < 0.05 and **p < 0.01.

RT-qPCR was used to validate results that had been obtained by RNA-Seq. From the list of 31 genes expressed differentially at the growth stage fruit development as a response to *L. botrana* herbivory at elevated CO_2_ and ambient CO_2_ concentrations (Supplementary Table S2), a set of 8 genes was selected for analysis, representing multiple modes of plants’ defence towards insect attack. A combination of two grapevine housekeeping genes (GADPH and cyclophilin), whose expression levels were relatively consistent, was found to be suitable as reference for normalization of gene expression (*M* = 0.800, CV = 0.275). Results show that except for one gene (pr10.3, which was also not classified as DEG in RNA-Seq analysis) all genes were significantly up-regulated or down-regulated (in the case of pr10.8) in grapevine plants as a response to *L. botrana* herbivory at elevated CO_2_ concentrations (Fig. 5, Supplementary Table S4), which is in perfect agreement to results obtained in RNA-Seq analysis (Supplementary Table S2). Differences in expression levels of five of the respective genes were still significant following the conservative Bonferroni correction (Supplementary Table S4). Three genes were also significantly up-regulated after *L. botrana* herbivory at ambient CO_2_ concentrations (Fig. 5, Supplementary Table S4), again confirming RNA-Seq data. In accordance to RNA-Seq data, no significant differences in gene expression levels were evident after *L. botrana* herbivory at the grapevine developmental stage berries ripe for harvest (Supplementary Table S5).

## Discussion

Albeit insects pose a significant threat to worldwide viticulture and are abundant members of vineyard ecosystems^30^, not much is known so far regarding herbivore-induced shifts in the grapevine transcriptome. In fact, for grapevine, only two studies have been published so far, which consider genome-wide transcriptional responses to herbivory of insects with a piercing-sucking mode of feeding, one assessing the transcriptional response of grapevine to the leaf galling stage of grapevine phylloxera, *Daktulosphaira vitifoliae*^31^, the other to feeding of the vine mealybug, *Planococcus ficus*^32^. In addition, a global proteomic study of the mesocarp and exocarp of field collected grape berries with visible signs of *L. botrana* feeding was conducted by Melo-Braga *et al*.^33^. It is well known that plants show local and systemic responses to attack by herbivorous insects and that intensity and chemical nature of these responses can be similar or different e.g. in different subspecies of the same host plant^34^. Our experimental design permitted an assessment only of systemic responses of grapevine plants to *L. botrana* herbivory. An aim of this study was to compare gene expression in response to herbivory during the grapevine phenological cycle. We therefore decided to sample similar grapevine organs, in this case those leaves, which were nearest to the *L. botrana* feeding site at two grapevine phenological stages (fruit development and berries ripe for harvest). However, so far nothing is known about a possible correlation between defence compound levels or signalling mechanisms in grape berries and leaves in response to *L. botrana* herbivory. Yet, at the systemic level, our study indicates for the first time that the grapevine transcriptional response to *L. botrana* herbivory is different if larvae had fed on very young berries (fruits beginning to develop) or harvest-ripe berries, respectively. However, RNA-Seq analysis showed no differences between grapevine plants that had been exposed to *L. botrana* feeding and/or elevated CO_2_ concentrations when obtained *p*-values were adjusted using Benjamini-Hochberg corrections. Significant differences between the different treatments were only obtained when less stringent criteria i.e. uncorrected *p*-values were used. However, RT-qPCR of a subset of these herbivory responsive genes showed significantly stronger expression levels as a result of *L. botrana* herbivory at elevated CO_2_ concentrations, with expression levels of five genes still being significantly different after a conservative Bonferroni correction.

In addition, *L. botrana* feeding on berries, which were ripe for harvest, resulted in only a very weak systemic transcriptional response compared to feeding on early developing fruits. It could be speculated whether this observation is related to an overall increase in resistance during grapevine seasonal development or rather to a senescence-related shut-down of the grapevine defence system close to the harvest period. In line with this, ontogenic resistance mechanisms related to herbivory have been described for a variety of plants^35,36^.

A second aim of this study was to assess if grapevine plants show a differential transcriptomic response to *L. botrana* herbivory under ambient and elevated CO_2_ concentrations and if elevated CO_2_ influences the plant’s response to herbivory on a transcriptomic level. We clearly showed that more transcripts were differentially expressed in grapevine plants as a response to *L. botrana* herbivory under eCO_2_ compared to aCO_2_ concentrations. Those transcripts that showed similar expression patterns under ambient and elevated CO_2_ concentrations after *L. botrana* herbivory in general had a higher fold-change of gene expression under eCO_2_. Accordingly, grapevine plants show a CO_2_ effect in response to *L. botrana* herbivory at the level of gene expression, with a much stronger overall transcriptomic response under future eCO_2_ conditions. Whether this general effect translates into a higher or lower susceptibility of grapevine towards *L. botrana* feeding under future CO_2_ concentrations, however, depends on the biological role of respective genes showing altered gene expression patterns.

Plants do not have adaptive immunity mechanisms but react with multiple layers of defence towards insect attack. The first perception of herbivores by the plant under attack is based on the recognition of insect oral secretions, of components of their mouth parts or of signals from injured plant cells^4,37,38^. These herbivore-associated elicitors (HAEs), herbivore-associated molecular patterns (HAMPs) or damage-associated molecular patterns (DAMPs) induce early signalling responses, such as fluctuations in cytosolic calcium concentration, production of reactive oxygen species (ROS) and elevated activity of mitogen activated protein kinases (MAPKs). These signalling cascades, in turn, activate transcription factors and dynamics of phytohormones, in particular ethylene (ET), jasmonate (JA) and salicylic acid (SA) stress hormone accumulation, eventually resulting in a transcriptional reconfiguration of metabolism (for reviews see^2,4,39^). Recent studies have indicated that ET as well as JA and SA signalling pathways are influenced by elevated CO_2_ concentrations, with an overall higher vulnerability of plants grown under elevated CO_2_ concentrations to insect damage (for a recent review see^15^).

In our study, we have identified changes in the grapevine’s transcriptome in each of these layers. In Brassicaceae the brassinosteroid insensitive 1-associated receptor kinase 1-like has been shown to act in pathogen-associated molecular pattern (PAMP)-triggered immunity and is involved in programmed cell death control^40,41^. Evidence is also accumulating that brassinosteroids play an important role in herbivore resistance^39^. A homologue of this protein was significantly upregulated in our study as a response to herbivory at both ambient and elevated CO_2_ concentrations and can thus be assumed to be also involved in HAMP related signalling in the grapevine – *L. botrana* system. Calcium-dependent protein kinases, calcium-binding proteins and a calmodulin-like protein all involved in calcium signalling and known to be implicated in a variety of plants’ responses to pathogen and herbivore attack including grapevine^42,43^ were significantly upregulated in grapevine leaves after *L. botrana* herbivory on young developing fruits. The same set of genes was found to be significantly upregulated in grapevine leaves as a result of vine mealybug *P. ficus* feeding^32^. Similarly, an increase in Lys-acetylation in the calcium binding protein CML was identified in the mesocarp and exocarp of grape berries after *L. botrana* feeding^33^, indicating that calcium signalling is an important component of grapevine defence against *L. botrana* larval herbivory.

Moreover, disease resistance proteins like RPM1 were significantly upregulated after herbivory. Homologues of these genes have been shown to confer resistance against a bacterial disease in *Arabidopsis*^44^ and play a critical role in protecting grapevine against infection by the downy mildew pathogen *Plasmopara viticola* via signalling pathways involving these molecules^45^. Another grapevine key defence-signalling gene that was found to be significantly upregulated as a response to *L. botrana* herbivory under both CO_2_ conditions is Enhanced Disease Susceptibility1 (EDS1). In grapevine, differences in expression levels of EDS1 are correlated with differential susceptibility towards the grape powdery mildew pathogen *Erysiphe necator*^46^. Moreover, expression of EDS1 has been shown to be also induced by salicylic acid (SA) and methyl salicylate (MeSA) treatments^47^.

A couple of genes residing at genetic loci known as MLO (Mildew Locus O) were significantly upregulated in grapevine leaves after *L. botrana* herbivory. MLO is a susceptibility factor required by adapted powdery mildew pathogens for host cell entry^48^. Resistance to grapevine powdery mildew pathogen *E. necator* can be achieved by knocking out these susceptibility genes^49^. Their role in grapevine susceptibility or resistance to insect attack remains yet to be shown.

Pathogenesis-related (PR) genes are a family of diverse proteins that have been widely proved to be involved in defence responses against pathogenic microorganisms in many plants including grapevine (for review see^50^). They are typically induced upon infection or herbivory^32,33^. Interestingly, the two PR proteins identified in this study to be regulated after *L. botrana* herbivory showed a different expression under ambient and elevated CO_2_, respectively. While PR 10.3 was significantly upregulated only under ambient CO_2_, PR 10.8 showed an opposite transcription at elevated CO_2_. This indicates that expression of various PR proteins as a response to herbivory might differ depending on atmospheric CO_2_ level and that future elevated CO_2_ concentrations might cause a shift in their expression patterns. However, as berries were exposed for a period of four days to *L. botrana* herbivory, secondary infections by fungal pathogens could as well be responsible for some of the differential expressions of PR proteins observed in our transcriptome analysis. Although there is some overlap between the defence response of plants against herbivores and those against pathogens, pathways are not identical and can even be antagonistic. Whether grapevine susceptibility or tolerance towards a variety of pests and diseases will differ in the future therefore requires further proof-of-concept field studies in FACE facilities.

In addition, expression of lipoxygenases and an allene oxide cyclase involved in JA biosynthesis were significantly upregulated after herbivory only at elevated CO_2_ concentrations. In soybean higher transcript levels of allene oxide cyclase were assumed to be involved in expression of strong resistance against the herbivorous lepidopteran insect *Helicoverpa armigera*^51^. Accordingly, future elevated CO_2_ concentrations might affect levels of *L. botrana* – grapevine interactions. In case of lipoxygenases and allene oxide cyclase, this might result in higher grapevine resistance levels, however, this assumption warrants further studies.

A strong CO_2_ effect was evident in grapevine leaves after *L. botrana* herbivory on ripening grape berries with a downregulation of expression of ethylene-responsive factors under elevated compared to ambient CO_2_ concentrations. Ethylene-responsive transcription factors are induced by elevated ethylene production as well as JA and activate the expression of defence-related genes and components of stress signal transduction pathways^52,53^. They are also involved in the activation of plant defence responses against insect herbivory. For example, olive (*Olea europaea* ) fruits infested with olive fly (*Bactrocera oleae* ) larvae showed a significant upregulation of several putative ethylene-responsive transcription factors^54^. Accordingly, a downregulation of their expression at higher atmospheric CO_2_ concentrations might indicate a higher susceptibility of grapevine plants to insect attack (herbivory). Since we found a differential regulation of these transcription factors only at the grapevine berry ripening stage, it could be assumed that this difference is rather related to the ripening process than to herbivory. Several hormones, including ethylene, control the process of grape berry ripening, however, grapevine is in general regarded as non-climacteric with only a slight increase of ethylene production related to the ripening process^55,56^. Yet, at this stage, we cannot completely rule out a possible effect of differences in ethylene production under ambient and elevated CO_2_ related to berry ripening being responsible for the different expression of ethylene-responsive transcription factors.

Finally, a gene involved in abscisic acid (ABA) binding and thus in ABA-signalling pathways, the major allergen Pru av1, was significantly down-regulated under *L. botrana* herbivory at eCO_2_ compared to aCO_2_ . Enhanced levels of ABA at increasing CO_2_ concentrations may result in a weakening of plant defence reactions as has been shown e.g. for *Arabidopsis thaliana* and the foliar pathogen *Pseudomonas syringae* pv. *tomato*^57^. If the same effect is evident in the case of herbivory remains to be shown.

## Conclusions

One of the key questions crop growers are facing in the future is if key pests will decrease or increase in their population density, abundance and damage potential and how plant protection strategies should be adapted accordingly. Our study has shown that future elevated CO_2_ concentrations will affect interactions between grapevine plants and one of its key insect pests, *L. botrana* larvae, with a differential expression of genes implemented at various stages of the grapevine defence system. How these transcriptomic changes translate into increased or decreased susceptibility or tolerance needs further research attention. Moreover, we have only assessed the effects of a single abiotic factor (CO_2_ concentration) on the grapevine – *L. botrana* system. However, future climate change will include multiple and combined stresses such as elevated temperatures and/or increasing drought stress. Further experiments under field conditions should be directed towards a combination of stressors and their effects on both the host crop plant as well as the herbivore pest insect with the aim to model and forecast future pest outbreaks.

## Methods

### VineyardFACE design

The Geisenheim VineyardFACE facility is located at Geisenheim University, Germany (49°59′N, 7°57′E; 96 m above sea level) in the German grapevine growing region Rheingau on the banks of river Rhine. Geisenheim has a temperate oceanic climate (Köppen-Geiger classification: Cfb) with mild winters and warm summers. The 30-year mean annual temperature of 1981–2010 period is 10.5 °C and total annual precipitation averages 543.1 mm. The soil at the experimental site is characterized as low-carbonate loamy sand to sandy loam.

The VineyardFACE was established in 2011 and consists of six ring-frame structures each with an inner diameter of 12 m, of which three are under elevated CO_2_ (eCO_2_) and three under ambient CO_2_ (aCO_2_) concentration. Each ring structure consists of 36 jets mounted at a height of 2.5 m equipped with fans to allow a force-free pre-dilution of the CO_2_. The operation of the fans and CO_2_-releasing valves is connected to wind speed and wind direction transmitters, which are installed at each eCO_2_ ring at 3 m height and distribute the released CO_2_ over the area through the wind movement. Hence, apart from the CO_2_ release, the microclimate within the grapevine canopy remains undisturbed in both the eCO_2_ and aCO_2_ rings alike. An aerial view and a schematic illustration of the Geisenheim VineyardFACE can be found as Supplementary Fig. S1. During the experiments described here, CO_2_ concentrations were measured by using two LI-8100 analyser control units installed at two heights (1.7 m and 0.75 m) in the grapevine canopy. Within aCO_2_ rings, an average level of 394 ± 0.4 ppm at 1.7 m height and 395 ± 0.4 ppm at 0.75 m height was reached between July and September 2015, while in eCO_2_ rings air was enriched during daylight hours to approximately 15–18% above the ambient CO_2_ (446 ± 9.4 ppm at 1.7 m height and 460 ± 12 ppm at 0.75 m height), which is the concentration predicted for the mid-21st century. Supplementary Fig. S2 illustrates CO_2_ concentrations in aCO_2_ and eCO_2_ rings during the course of the experiments (mid-July: Supplementary Fig. S2a and end of September 2015: Supplementary Fig. S2b) described here. Data of weather conditions during the experimental periods are provided in Supplementary Table S6.

Within VineyardFACE rings, vines *Vitis vinifera* L. cv. Riesling (clone 198–30 Gm) grafted on rootstock SO4 (clone 47 Gm) and cv. Cabernet Sauvignon grafted on rootstock 161–49, respectively, were planted in April 2012 as one year old potted plants. Each ring contains seven rows of cv. Riesling and cv. Cabernet Sauvignon grapevine plants, which were planted alternately across a central divide (Supplementary Fig. S1). Vines were planted with a spacing of 0.9 m within rows and 1.8 m between rows, with a north-south orientation. Using a vertical shoot positioning (VSP)-type trellis system canes were pruned to 5 nodes per m^2^. Management of the vineyard was according to the principles of good agricultural praxis (GAP) and integrated pest management (IPM) in viticulture. Cover crop consisted of Freudenberger WB 130 mixture and was administered to every second row, while every other second row was ploughed once in spring and was largely bare or covered with spontaneous vegetation. Grapevines were bearing fruits for the first time in fall 2013. Field experiments described here were performed only on cv. Riesling vines.

### Insects

Experiments were conducted with *L. botrana* larvae derived from an inbred laboratory strain maintained at Geisenheim University, Geisenheim, Germany. Larvae were cultured in groups in plastic boxes (20 × 15 cm and 9 cm high) in an insect rearing room (24 ± 1 °C; 40 ± 12% relative humidity; light/dark photoperiod: 16:8 h) and were fed *ad libitum* with a modified semi-synthetic diet according to the general-purpose diet of Singh^58^. Briefly, agar and alfalfa sprouts were mixed and boiled and sucrose, yeast, wheat germ, cholesterol, casein, sunflower oil and Wesson’s salt mixture were added. Vitamin mixture, sorbic acid, propionic acid and 95% ethanol were mixed separately and added to the diet after cooling. Larvae were cultured until they reached the 2^nd^ larval instar stage, when they were used in experiments described below.

### Field experiments, sample collection and total RNA extraction

Overall, three different factors were considered in field experiments, i.e. (1) two CO_2_ concentrations; (2) with and without *L. botrana* herbivory; (3) two grapevine growth stages. Accordingly, field experiments were conducted at two periods in mid-July and end of September 2015, respectively, covering two different principal grapevine growth stages, i.e. growth stage “development of fruits” (phenological stage “berries pea-sized”; BBCH 75) and growth stage “ripening of berries” (phenological stage “berries ripe for harvest”; BBCH 89)^59^. At each time point, three vines in each ring were infested with *L. botrana* larvae and other three vines were used as control plants (non-infested), resulting in 9 biological replicates for infested and 9 for non-infested grapevine plants for each time point of sampling and for aCO_2_ and eCO_2_, respectively. Non-infested control plants at fruit development and berries harvest-ripe stage are designated as aC-f and aC-b for plants grown under aCO_2_ and as eC-f and eC-b for those under eCO_2,_ respectively. Similarly, plants exposed to *L. botrana* herbivory at fruit development and berries harvest-ripe stage each are tagged as aLb-f and aLb-b (grown under aCO_2_) and as eLb-f and eLb-b (grown under eCO_2_), respectively. Supplementary Table S7 summarizes the different treatments and the respective research question.

Prior to the experiments, *L. botrana* larvae in their second instar were starved for 1 day in the laboratory. Accordingly, five *L. botrana* larvae were placed per grape bunch and were let to feed for four days. In order to prevent escape of larvae, nylon mesh bags (12 × 16 cm) were used to cover bunches. Control plants were treated in the same way except for infestation with larvae.

After four days of feeding, the nearest leaf to a *L. botrana* feeding site was collected both from infested and control plants, respectively and was immediately flash frozen in the field in liquid nitrogen, followed by storage at −80 °C until RNA extraction. Total RNA was extracted from 100 mg frozen leaf samples in the presence of liquid nitrogen using Spectrum^TM^ Plant Total RNA Kit (Sigma-Aldrich) according to manufacturer’s protocol. DNA was removed during extraction using On-column DNase I digestion (Sigma-Aldrich). RNA quantification was performed using a NanoDrop 1000 Spectrophotometer (Thermo Scientific, Wilmington, USA). After extraction, equivalent amounts of RNA from each of the three biological replicates obtained from infested and non-infested grapevine plants per VineyardFACE ring were pooled, respectively, resulting in three RNA pooled samples for each CO_2_ concentration (aCO_2_ and eCO_2_; with the respective three FACE rings as replicates), treatment (control plants and plants exposed to *L. botrana* herbivory) and growth stage (fruit development and berry ripening). A total of 1 μg of total RNA for each pool was ethanol precipitated and was sent to Macrogen Korea (Seoul, Korea) for RNA sequencing.

### RNA sequencing and bioinformatics analysis

Quantity and integrity of the extracted total RNA was determined using Agilent 2100 bioanalyzer (Agilent Technologies, USA), to be RIN >8. The cDNA library was constructed by Macrogen Korea using the TruSeq RNA Library Prep Kit v2 (Illumina) according to the manufacturer’s instructions. Briefly, the mRNA molecules containing poly-A tails were purified using oligo (dT) beads from the RNA samples. Purified mRNA transcripts were randomly fragmented and reverse transcribed into cDNA, onto which adapters were ligated on both ends. After PCR amplification, fragments with insert sizes between 200–400 bp were selected for paired-end sequencing using the Illumina HiSeq 4000 system.

Raw reads were filtered to remove adapter sequences, contaminant DNA and PCR duplicates using Trimmomatic 0.32 and high quality Illumina raw reads with Phred scores ≥30 were kept for assembly. Trimmed reads were mapped to the *V. vinifera* reference genome (GenBank accession number GCF_000003745.3) with TopHat version 2.0.12. After read mapping, Cufflinks version 2.21 was used for assembly of known transcripts, alternative splicing transcripts and novel transcripts. Expression profiles of assembled transcripts were calculated for each sample and gene expression counts were normalized using the fragments per kilobase transcript length per million fragments mapped (FPKM) value. Contigs with FPKM values of 0 were discarded. Euclidean distance was used to measure between samples dissimilarities over gene expression values and multidimensional scaling analysis (MDS) was performed with each sample’s log_2_(FPKM + 1) value. Differential expressed gene (DEG) analysis was accomplished between each pair of samples using conditions of fold change ≥2 and an independent t-test raw *p*-value < 0.05. In separate analyses, *p*-values were either left unadjusted or adjusted for multiple testing with the Benjamini and Hochberg method^60^. Gene Ontology (GO)^61^ and the Kyoto Encyclopedia of Genes and Genomes (KEGG)^62^ databases were used to identify pathway maps based on groups of annotated genes that are differentially expressed in a given pair of samples.

### Validation of RNA-Seq by RT-qPCR

Gene expression levels based on RNA-Seq data were validated using RT-qPCR with eight genes identified as DE under *L. botrana* herbivory at the growth stage fruit development: brassinosteroid insensitive 1-associated receptor kinase 1-like (cdpk1, Gene ID 100266543), calcium-binding allergen Ole e 8-like (cba8, Gene ID 100253496), enhanced disease susceptibility 1 (eds1, Gene ID: 100233033), disease resistance protein RPM1-like (drp1, Gene ID 100256051), mildew resistance locus o 10 (mlo10, Gene ID: 100233061), pathogenesis-related protein 10.3 (pr10.3, Gene ID 100267074), pathogenesis-related protein 10.8 (pr10.8, Gene ID 100258426) and allene oxide synthase (aos, Gene ID100267750). As housekeeping genes glyceraldehyde-3-phosphate dehydrogenase (GAPDH, Gene ID: 100233024) and cyclophilin (GenBank: EC969926) were used, which were previously identified as stable reference genes for RT-qPCR analysis in grapevine plant material^63^. Primer details are presented in Supplementary Table S8. For RT-qPCR independent biological RNA samples extracted from the same 24 grapevine plants as for RNA-Seq (Supplementary Table S1) were used, resulting in three biological replicates per treatment (herbivory and CO_2_ concentration). RT-qPCR was conducted using RevertAid First Strand cDNA Synthesis Kit (Thermo Scientific) and Maxima SYBR Green (Thermo Scientific) on an iQ5 Multicolor iCycler (Bio-Rad). Three technical replicates were run per biological sample for each gene. Normalized relative expression levels were calculated using the method implemented in qbase + Version 3.2 (Biogazelle). Reference genes were evaluated based on expression stability (*M* values) and coefficients of variation (CV) using qbase+. Statistical differences in pairwise comparisons of average relative fold expression levels were calculated using an unpaired t-test, with a *p* value of < 0.05 considered to be significant.

## Supplementary information

Supplementary Material

## Data Availability

The raw datasets generated during the current study are available in the NCBI Sequence Read Archive under BioProject ID PRJNA417047 and in Sequence Read Archives under accession numbers SAMN08093445 - SAMN08093492 (Supplementary Table S1). All other datasets analysed during the current study are either included in this published article (and its Supplementary Information files) or are available from the corresponding author on reasonable request.
