# Supplementary material for: Elevated atmospheric CO2 concentrations alter grapevine (Vitis vinifera) systemic transcriptional response to European grapevine moth (Lobesia botrana) herbivory
Source: Sci Rep. 2019 Feb 28;9:2995. doi: 10.1038/s41598-019-39979-5 (PMC6395777; doi:10.1038/s41598-019-39979-5)
Supplement: Supplementary file 1 — Supplementary Material [file 41598_2019_39979_MOESM1_ESM.docx]

Supporting Information

**Elevated atmospheric CO_2_ concentrations alter grapevine (*Vitis vinifera*) systemic transcriptional response to European grapevine (*Lobesia botrana*) herbivory**

**Annette Reineke*, Moustafa Selim**

Geisenheim University, Department of Crop Protection, Von-Lade-Str. 1, D-65366 Geisenheim, Germany

***Corresponding author:**

Annette Reineke

Geisenheim University, Department of Crop Protection

Von-Lade-Str. 1, D-65366 Geisenheim, Germany

Tel.: ++49-6722-502413

Fax.: ++49-6722-502410

email: [annette.reineke@hs-gm.de](mailto:annette.reineke@hs-gm.de)

Supplementary Table S1. RNA-Seq data statistics of 24 grapevine leaf samples obtained from (1) two different levels of CO_2_ concentration (ambient: a; elevated: e) and (2) exposed (Lb) or not exposed (C) to *L. botrana* herbivory at (3) two grapevine growth stages (fruit development: –f; berry ripening: –b). Three RNA pools (indicated by 1, 2, 3) representing 3 biological replicates (each from one FACE ring) for each CO_2_ concentration, treatment and growth stage were sequenced.

| **Sample ID** | **SRA accession** | **Total reads (raw)** | **Total reads (trimmed)** | **GC (%)** | **Overall read mapping ratio (%)** |
| --- | --- | --- | --- | --- | --- |
| aC-f1 | SAMN08093445 | 15,861,402 | 15,555,428 | 49.51 | 72.4 |
| aC-f2 | SAMN08093447 | 15,578,870 | 15,213,498 | 46.61 | 84.9 |
| aC-f3 | SAMN08093449 | 14,067,862 | 13,770,790 | 46.64 | 85.6 |
| eC-f1 | SAMN08093451 | 18,598,222 | 18,114,812 | 46.76 | 85.6 |
| eC-f2 | SAMN08093453 | 4,958,804 | 4,864,628 | 47.6 | 79.9 |
| eC-f3 | SAMN08093455 | 16,849,062 | 16,500,380 | 48.89 | 78.0 |
| aLb-f1 | SAMN08093457 | 17,016,456 | 16,702,712 | 48.78 | 70.0 |
| aLb-f2 | SAMN08093459 | 17,117,838 | 16,760,802 | 48.41 | 74.7 |
| aLb-f3 | SAMN08093461 | 14,647,170 | 14,334,176 | 46.96 | 83.3 |
| eLb-f1 | SAMN08093463 | 17,451,360 | 17,030,680 | 46.19 | 85.7 |
| eLb-f2 | SAMN08093465 | 15,676,426 | 15,342,104 | 47.24 | 83.8 |
| eLb-f3 | SAMN08093467 | 17,509,502 | 17,154,298 | 48.82 | 73.0 |
| aC-b1 | SAMN08093469 | 18,747,522 | 18,354,022 | 51.76 | 52.1 |
| aC-b2 | SAMN08093471 | 17,046,064 | 16,665,326 | 48.91 | 77.0 |
| aC-b3 | SAMN08093473 | 16,464,372 | 16,105,806 | 50.95 | 62.8 |
| eC-b1 | SAMN08093475 | 16,614,502 | 16,312,410 | 51.17 | 55.0 |
| eC-b2 | SAMN08093477 | 13,706,066 | 13,444,896 | 48.35 | 75.7 |
| eC-b3 | SAMN08093479 | 16,668,752 | 16,269,546 | 46.97 | 77.7 |
| aLb-b1 | SAMN08093481 | 17,324,706 | 16,978,112 | 50.32 | 60.6 |
| aLb-b2 | SAMN08093483 | 15,903,092 | 15,561,132 | 50.37 | 65.8 |
| aLb-b3 | SAMN08093485 | 14,584,348 | 14,217,714 | 49.73 | 68.4 |
| eLb-b1 | SAMN08093487 | 15,679,026 | 15,338,534 | 51.14 | 56.7 |
| eLb-b2 | SAMN08093489 | 12,970,936 | 12,690,658 | 48.85 | 71.9 |
| eLb-b3 | SAMN08093491 | 17,171,906 | 16,781,556 | 46.63 | 80.8 |

Supplementary Table S2. Genes involved in “plant-pathogen interaction”, “defence response” or “response to biotic stimuli” as identified by GO enrichment analysis or via KEGG database classification in grapevine plants (growth stage fruit development) as a response to *L. botrana* herbivory at elevated CO_2_ and ambient CO_2_ concentrations. IDs are according to GenBank entries, fold change of expression levels of genes in three pairwise comparisons is shown only for significant genes (*p* < 0.05). For sample codes see Supplementary Table S6. Expression of genes printed in bold letters was validated by RT-qPCR.

| **Gene_ID** | **Protein_ID** | **Description** | **Fold change** | | |
| --- | --- | --- | --- | --- | --- |
|  |  |  | **aLb-f vs. aC-f** | **eLb-f vs. eC-f** | **eLb-f vs. aLb-f** |
| **100266543** | **XP_010657614.1** | **brassinosteroid insensitive 1-associated receptor kinase 1-like** | **2.627** | **2.996** |  |
| 100243573 | XP_002278179.1 | calcium-dependent protein kinase 2 | **2.088** | **2.596** |  |
| 100248850 | XP_002267099.1 | calcium-dependent protein kinase 28 |  | **2.365** |  |
| **100253496** | **XP_002274476.1** | **calcium-binding allergen Ole e 8-like** | **2.533** | **4.695** |  |
| 100244152 | XP_002283755.1 | calmodulin-like protein 11 | **2.166** |  |  |
| 100255889 | XP_002282351.2 | caltractin | **2.062** |  |  |
| 100263254 | XP_010649201.1 | probable calcium-binding protein CML41 | **2.974** |  |  |
| 100267648 | XP_002273785.1 | endoplasmin homolog | **2.222** | **2.222** |  |
| **100233033** | **NP_001267967.1** | **enhanced disease susceptibility 1 (EDS1)** | **2.494** | **3.091** |  |
| 100250236 | XP_002283967.2 | lipase-like PAD4 | **6.804** | **6.970** |  |
| 100253457 | XP_002280786.1 | lipase-like PAD4 | **6.463** | **11.268** |  |
| 100257078 | XP_002280729.1 | lipase-like PAD4 | **7.493** | **10.771** |  |
| 100241155 | XP_002281195.1 | probable disease resistance protein At1g12280 | **2.109** | **2.195** |  |
| 104881039 | XP_010657950.1 | probable disease resistance protein At4g27220 | **2.376** | **2.924** |  |
| 100853252 | XP_010652660.1 | probable disease resistance protein At5g66900 |  | **3.037** |  |
| 100854398 | XP_010658280.1 | putative disease resistance protein At1g50180 |  | **-2.161** |  |
| 100244083 | XP_002276170.1 | protein SGT1 homolog |  | **2.107** |  |
| 100252764 | XP_002268128.2 | uncharacterized aarF domain-containing protein kinase At1g79600, chloroplastic-like |  | **-2.037** |  |
| 100854803 | XP_003633333.1 | disease resistance protein RPM1-like |  | **2.044** |  |
| **100256051** | **XP_002275317.3** | **disease resistance protein RPM1-like** | **3.582** | **3.055** |  |
| 100852949 | XP_003632405.1 | disease resistance protein RPM1-like | **2.058** | **2.540** |  |
| 100267151 | XP_010657373.1 | G-type lectin S-receptor-like serine/threonine-protein kinase At4g03230 | **2.6289** | **2.225** |  |
| 100246525 | XP_002274785.1 | major allergen Pru av 1 | **3.414** |  | **-2.045** |
| 100261887 | XP_002274535.1 | major allergen Pru av 1 | **2.661** |  | **-2.814** |
| 100256795 | XP_002274617.1 | major allergen Pru av 1 |  |  | **-2.796** |
| 100233053 | NP_001267997.1 | mildew resistance locus o 15 (MLO15) | **2.362** | **2.524** |  |
| 100233063 | NP_001268013.1 | MLO11 protein | **3.274** | **3.051** |  |
| **100233061** | **XP_002276608.1** | **MLO-like protein 10** | **2.225** | **2.362** |  |
| **100267074** | **NP_001267956.1** | **pathogenesis-related protein 10.3** | **3.891** |  |  |
| **100258426** | **XP_002273815.2** | **pathogenesis-related protein 10.8** |  | **-3.257** |  |
| **100267750** | **XP_002283780.1** | **allene oxide synthase** |  | **2.707** |  |

Supplementary Table S3. Genes identified by GO enrichment analysis in grapevine plants (growth stage berry ripening) as a response to *L. botrana* herbivory at elevated CO_2_ and ambient CO_2_ concentrations. IDs are according to GenBank entries, fold change of expression levels of genes in two pairwise comparisons is shown only for significant genes (*p* < 0.05). For sample codes see Supplementary Table S6.

| **GO Term(s)** | **Gene_ID** | **Protein_ID** | **Description** | **Fold change** | |
| --- | --- | --- | --- | --- | --- |
|  |  |  |  | **eLb-b vs. eC-b** | **eLb-b vs. aLb-b** |
| GO:0003700 | 100247374 | XP_002276500.1 | transcription factor BEE 3 | **2.272** |  |
| GO:0003700 | 100264451 | XP_002279570.1 | zinc finger protein ZAT10 | **-2.186** |  |
| GO:0003677 | 100265533 | XP_002284384.1 | zinc finger protein ZAT11 |  | **-2.636** |
| GO:0003700 | 100241331 | XP_010653659.1 | transcription elongation factor SPT6 |  | **2.133** |
| GO:0003700; GO:0003677 | 100242144 | XP_002276215.1 | ethylene-responsive transcription factor 4 |  | **-2.254** |
| GO:0003700; GO:0003677 | 100243417 | XP_002279760.1 | ethylene-responsive transcription factor 12 |  | **-2.164** |
| GO:0003700; GO:0003677 | 100254640 | XP_002270581.2 | ethylene-responsive transcription factor 2 |  | **-2.031** |

Supplementary Table S4. Expression of eight genes involved in grapevine’s response (growth stage fruit development) to *L. botrana* herbivory at elevated CO_2_ and ambient CO_2_ concentrations. Expressions were assessed by RT-qPCR in three biological replicates per treatment (herbivory and control) and CO_2_ concentration. The normalized relative fold change of expression levels in three pairwise comparisons is shown with the 95% confidence interval. Significant differences in expression ratios at *p* < 0.05 are indicated by an asterisk. Significant differences in expression ratios after Bonferroni corrections are indicated by two asterisks. For sample codes see Supplementary Table S6.

|  |  | **Ratio** | | |
| --- | --- | --- | --- | --- |
| **Gene ID** | **Gene** | **aLb-f vs. aC-f (95% CI)** | **eLb-f vs. eC-f (95% CI)** | **aLb-f vs. eLb-f (95% CI)** |
| 100266543 | brassinosteroid insensitive 1-associated receptor kinase 1-like (cdpk1) | 1.68 (0.5; 5.7) | 2.40 (1.6; 3.6) * | 0.78 (0.3; 2.5) |
| 100253496 | calcium-binding allergen Ole e 8-like (cba8) | 2.91 (0.9; 9.4) | 5.36 (2.8; 1.0) ** | 0.73 (0.2; 2.4) |
| 100233033 | enhanced disease susceptibility 1 (eds1) | 2.48 (0.8; 7.3) | 3.46 (1.7; 7.2) * | 0.90 (0.3; 2.7) |
| 100256051 | disease resistance protein RPM1-like (drp1) | 1.87 (0.4; 8.1) | 2.59 (2.1; 3.2) ** | 0.82 (0.2; 3.5) |
| 100233061 | mildew resistance locus o 10 (mlo10) | 3.06 (1.8; 5.3) * | 3.16 (2.2; 4.6) ** | 0.94 (0.7; 1.3) |
| 100267074 | pathogenesis-related protein 10.3 (pr10.3) | 1.02 (2.4; 4.3) * | 3.95 (5.8; 2.7) | 1.8 (0.3; 1.2) |
| 100258426 | pathogenesis-related protein 10.8 (pr10.8) | 0.60 (1.2; 2.9) | 4.7 (3.6; 6.1) ** | 1.2 (0.2; 6.8) |
| 100267750 | allene oxide synthase (aos) | 6.82 (2.9; 1.6) * | 4.09 (2.9; 5.8) ** | 1.57 (0.9; 2.8) |

Supplementary Table S5. Expression of eight genes involved in grapevine’s response (growth stage berry ripe for harvest) to *L. botrana* herbivory at elevated CO_2_ and ambient CO_2_ concentrations. Expressions were assessed by RT-qPCR in three biological replicates per treatment (herbivory and control) and CO_2_ concentration. The normalized relative fold change of expression levels in three pairwise comparisons is shown with the 95% confidence interval. No significant differences in expression ratios at *p* < 0.05 were evident. For sample codes see Supplementary Table S6.

|  |  | **Ratio** | | |
| --- | --- | --- | --- | --- |
| **Gene ID** | **Gene** | **aLb-b vs. aC-b (95% CI)** | **eLb-b vs. eC-b (95% CI)** | **aLb-b vs. eLb-b (95% CI)** |
| 100266543 | brassinosteroid insensitive 1-associated receptor kinase 1-like (cdpk1) | 1.01 (0.7; 1.5) | 1.14 (0.7; 1,8) | 0.76 (0.5; 1.1) |
| 100253496 | calcium-binding allergen Ole e 8-like (cba8) | 1.08 (0.4; 2.9) | 1.54 (0.7; 3.2) | 0.63 (0.3; 1.3) |
| 100233033 | enhanced disease susceptibility 1 (eds1) | 0.85 (0.4; 1.8) | 1.29 (0.6; 2.7) | 0.61 (0.2; 1.3) |
| 100256051 | disease resistance protein RPM1-like (drp1) | 0.79 (0.2; 2.5) | 1.10 (0.6; 1.9) | 0.59 (0.2; 1.7) |
| 100233061 | mildew resistance locus o 10 (mlo10) | 0.78 (0.4; 1.5) | 1,11 (0.9; 1.4) | 0.84 (0.6; 1.1) |
| 100267074 | pathogenesis-related protein 10.3 (pr10.3) | 1.0 (0.3; 3.9) | 1.07 (0.3; 3.6) | 0.99 (0.2; 4.0) |
| 100258426 | pathogenesis-related protein 10.8 (pr10.8) | 0.5 (0.05; 5.6) | 0.90 (0.2; 3.3) | 1.44 (0.5; 3.7) |
| 100267750 | allene oxide synthase (aos) | 0.80 (0.3; 1.8) | 1.54 (0.8; 2.9) | 0.47 (0.2; 1.1) |

Supplementary Table S6. Weather conditions during the experimental periods conducted in the Geisenheim VineyardFACE facility in July and September 2015.

| **Date 2015** | **Air temperature [°C]** | | | **Soil temperature [°C]** | | | | | **Precipitation** | **Evaporation** | **Sunshine** |
| --- | --- | --- | --- | --- | --- | --- | --- | --- | --- | --- | --- |
|  | Daily average | Max. | Min. | Daily average 5 cm | Daily average 10 cm | Daily average 20 cm | Daily average 50 cm | Daily average 100 cm | Daily sum [mm] | Haude - Gras [mm] | Daily sum [hours] |
| 15. Jul | 22.6 | 29.4 | 16.1 | 26.7 | 26.1 | 24.6 | 22.6 | 21.1 | 0.0 | 5.8 | 9.6 |
| 16. Jul | 25.4 | 32.6 | 18.0 | 29.5 | 28.6 | 26.8 | 23.7 | 21.1 | 0.0 | 8.3 | 13.7 |
| 17. Jul | 27.3 | 35.3 | 20.0 | 29.4 | 28.7 | 27.5 | 24.7 | 21.4 | 0.8 | 11.2 | 8.9 |
| 18. Jul | 25.0 | 31.1 | 19.2 | 29.2 | 28.8 | 27.8 | 25.3 | 21.8 | 0.0 | 7.5 | 8.6 |
|  |  |  |  |  |  |  |  |  |  |  |  |
|  |  |  |  |  |  |  |  |  |  |  |  |
| 27. Sep | 13.9 | 18.8 | 9.2 | 13.6 | 14.0 | 14.7 | 15.5 | 16.3 | 0.0 | 3.0 | 9.0 |
| 28. Sep | 12.8 | 18.3 | 6.5 | 12.6 | 13.0 | 13.8 | 15.1 | 16.2 | 0.0 | 2.8 | 10.2 |
| 29. Sep | 13.4 | 18.9 | 6.9 | 12.6 | 12.9 | 13.7 | 14.8 | 16.1 | 0.0 | 2.9 | 8.0 |
| 30. Sep | 13.3 | 17.9 | 9.6 | 13.2 | 13.5 | 14.0 | 14.8 | 15.9 | 0.0 | 2.8 | 10.5 |

Supplementary Table S7. Description of grapevine treatments (two CO_2_ concentrations, with and without *L. botrana* herbivory, two different grapevine phenological stages), and the resulting pairs of comparison with respective research questions.

| **Code** | **CO_2_ concentration** | **Herbivory** | **Grapevine phenological stage** |
| --- | --- | --- | --- |
| aC-f | ambient (ca. 400 ppm) | no | fruit development |
| aC-b |  |  | berries ripe for harvest |
| eC-f | elevated (ca. 450 ppm) | no | fruit development |
| eC-b |  |  | berries ripe for harvest |
| aLb-f | ambient (ca. 400 ppm) | yes | fruit development |
| aLb-b |  |  | berries ripe for harvest |
| eLB-f | elevated (ca. 450 ppm) | yes | fruit development |
| eLb-b |  |  | berries ripe for harvest |
|  |  | | |
| **Pair** | **Question** | | |
| aC-f vs. aLb-f/ aC-b vs. aLb-b | How do grapevine plants respond to *L. botrana* herbivory under ambient CO_2_ concentration (at growth stages fruit development or berry ripening)? | | |
| eC-f vs. eLb-f/ eC-b vs. eLb-b | How do grapevine plants respond to *L. botrana* herbivory under elevated CO_2_ concentration (at growth stages fruit development or berry ripening)? | | |
| aC-f vs. eC-f/ aC-b vs. eC-b | How do grapevine plants respond to elevated CO_2_ concentration (at growth stages fruit development or berry ripening)? | | |
| aLb-f vs. eLb-f/ aLb-b vs. eLb-b | How do grapevine plants respond to elevated CO_2_ concentration under *L. botrana* herbivory (at growth stages fruit development or berry ripening)? | | |

Supplementary Table S8. Primer information for grapevine *L. botrana* herbivory responsive genes and two housekeeping genes (GADPH and cyclophilin) used for validation of RNA-Seq data by RT-qPCR.

| **Gene ID** | **Gene** | **Primer Sequence Forward** | **Primer Sequence Reverse** | **Amplicon (bp)** | **Efficiency (%)** | **R^2^** |
| --- | --- | --- | --- | --- | --- | --- |
| 100266543 | brassinosteroid insen-sitive 1-associated receptor kinase 1-like (cdpk1) | 5’-AGGGGGCTTGCTTATTTGCATG-3’ | 5’-ATGCTTGCAGCCTTCACATCAC-3’ | 71 | 104.6 | 0.995 |
| 100253496 | calcium-binding aller-gen Ole e 8-like (cba8) | 5’-AGTTGCACGCGGTGTTCAAG-3’ | 5’-ACTCCTCGAAATTGACGCAACC-3’ | 111 | 108.5 | 0.966 |
| 100233033 | enhanced disease susceptibility 1 (eds1) | 5’-ACCCGTGGCTTTGCTATT-3’ | 5’-GGAAAGGTACTAGCATCCATCTT-3’ | 104 | 95.7 | 0.998 |
| 100256051 | disease resistance protein RPM1 (drp1) | 5’-AGTTCGCACAGAAGCTTATGCC-3’ | 5’-TGCTTAATGATCGCGAGCCTTG-3’ | 150 | 100.5 | 0.994 |
| 100233061 | mildew resistance locus o 10 (mlo10) | 5’-GGGCATCCATAATTCCCTTAGT-3’ | 5’-GGACCACTGCATGTCTTTCT-3’ | 105 | 85.4 | 0.986 |
| 100267074 | pathogenesis-related protein 10.3 (pr10.3) | 5’-GATGTTTTGACGAGCGGCATTG-3’ | 5’-TCTTTGCCGCCCTTAACGTG-3’ | 149 | 114.0 | 0.986 |
| 100258426 | pathogenesis-related protein 10.8 (pr10.8) | 5’-ATGGGTGTCACCAGTTTCACAC-3’ | 5’-AGCTTTGGGATCAGGTTGTTGG-3’ | 101 | 80.1 | 0.950 |
| 100267750 | allene oxide synthase (aos) | 5’-TTGCCACAACCTCCTTTTCGC-3’ | 5’-ATTGGGTGTGCAGTTTCACTCC-3’ | 113 | 108.9 | 0.966 |
| 100233024 | GAPDH | 5’-TCAAGGTCAAGGACTCTAACACC-3’ | 5’-CCAACAACGAACATAGGAGCA-3’ | 226 | 104.3 | 0.999 |
| EC969926 | cyclophilin | 5’-GGAGCCTGAGCCTACCTTCTC-3’ | 5’-GTGTTCGGCCAGGTGGTAGA-3’ | 66 | 99.7 | 0.989 |


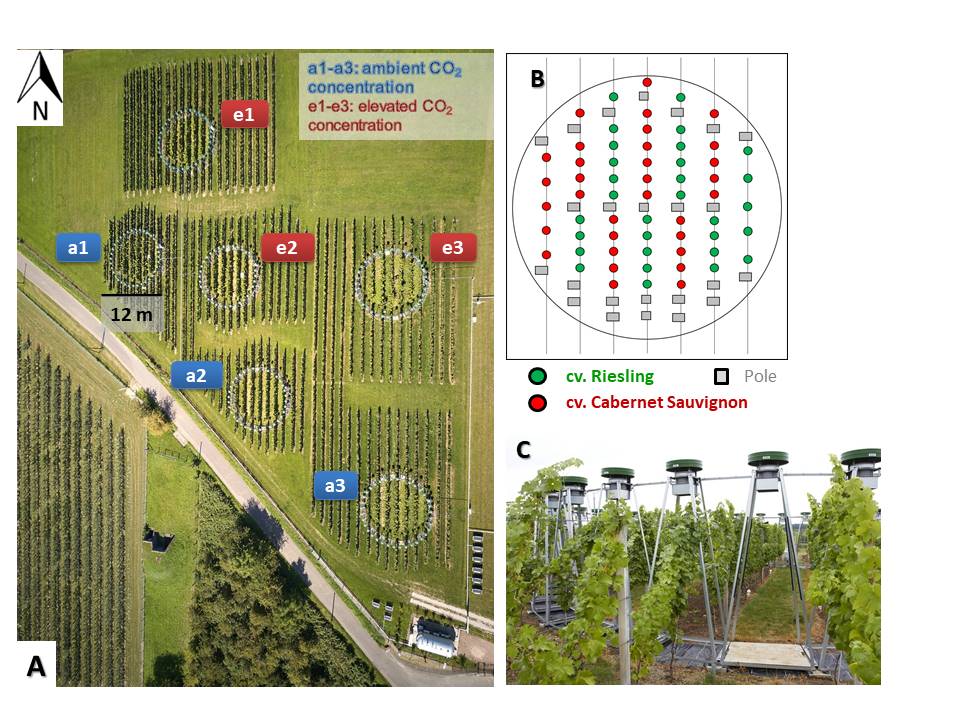


Supplementary Fig. S1: (A) Aerial view (picture by Winfried Schönbach) of the Geisenheim VineyardFACE experimental site showing localization of the three ambient CO_2_ (designated as a1, a2 and a3) and three elevated CO_2_ (designated as e1, e2 and e3) grapevine FACE rings. One ring has a diameter of 12 m. (B) Schematic illustration of one VineyardFACE ring planted with two different grapevine varieties (green dots: cv. Riesling and red dots: cv. Cabernet Sauvignon). Distance between rows is 1.80 m and between vines 0.90 m. (C) Detail of the Geisenheim VineyardFACE facility (picture by Winfried Schönbach).


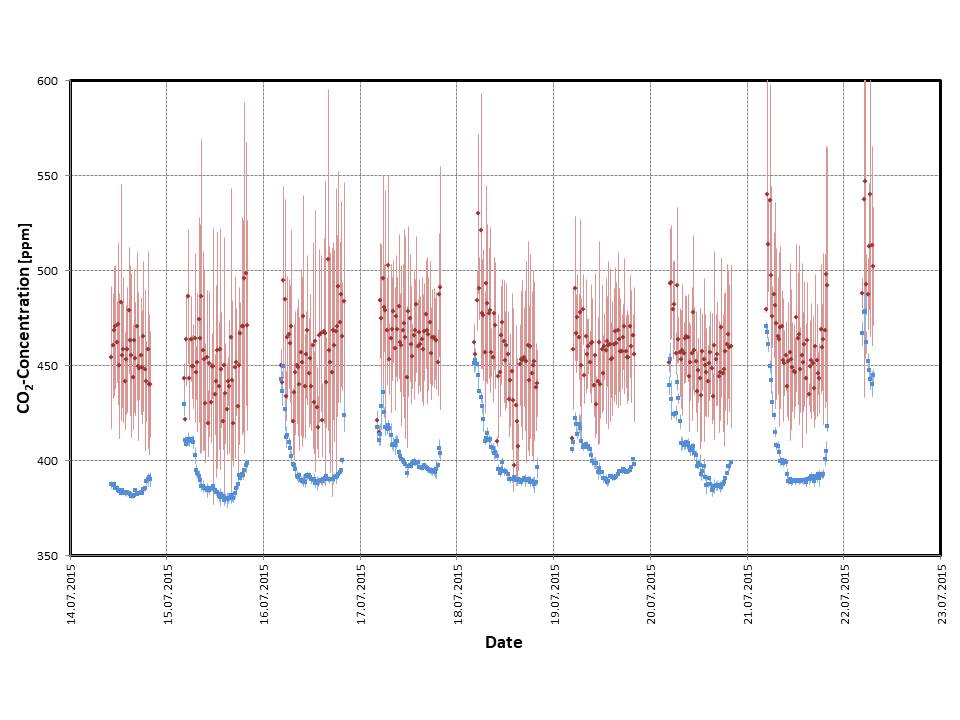
Supplementary Fig. S2a: Daily CO_2_ concentration measured in July 2015 at a height of 1.7 m in Geisenheim VineyardFACE rings with ambient (blue dots) and elevated (red dots) CO_2_ concentration including standard error. CO_2_ concentrations were measured in 20 min intervals between 4:20 and 20:00.


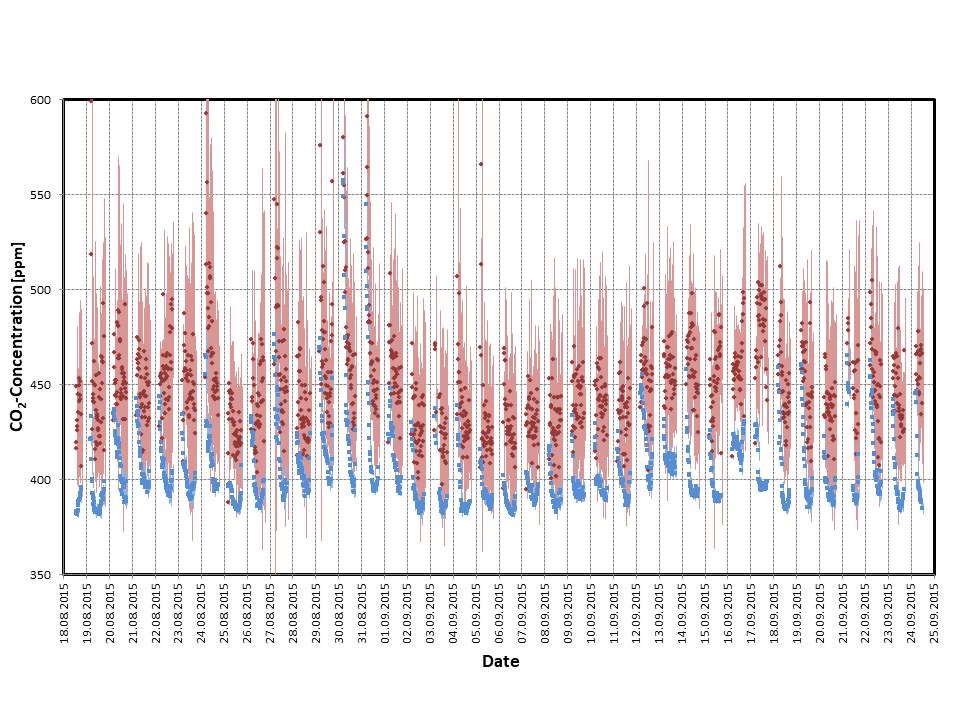
Supplementary Fig. S2b: Daily CO_2_ concentration measured from mid-August to end of September 2015 at a height of 1.7 m in Geisenheim VineyardFACE rings with ambient (blue dots) and elevated (red dots) CO_2_ concentration including standard error. CO_2_ concentration was measured in 20 min intervals between 5:20 and 19:00.
